# Artificial intelligence-based optimization for extracellular L-glutaminase free L-asparaginase production by *Streptomyces violaceoruber* under solid state fermentation conditions

**DOI:** 10.1038/s41598-024-77867-9

**Published:** 2024-11-28

**Authors:** Noura El-Ahmady El-Naggar, Ragaa A. Hamouda, Naglaa Elshafey

**Affiliations:** 1 Department of Bioprocess Development, Genetic Engineering and Biotechnology Research Institute, City of Scientific Research and Technological Applications (SRTA-City), New Borg El-Arab City, 21934 Alexandria Egypt; 2 Microbial Biotechnology Department, Genetic Engineering and Biotechnology Research Institute, University of Sadat City, Sadat City, Egypt; 3 Department of Applied Radiologic Technology, College of Applied Medical Sciences, University of Jeddah, Jeddah, 23218 Saudi Arabia; 4 Botany and Microbiology Department, Faculty of Science, Arish University, Al-Arish, 45511 Egypt

**Keywords:** L-asparaginase; *Streptomyces*; Identification, Optimization, Central composite design, Artificial neural network, Applied microbiology, Bacteria

## Abstract

The bacterial L-asparaginase is a highly effective chemotherapeutic drug and a cornerstone of treatment protocols used for treatment the acute lymphoblastic leukemia in pediatric oncology. A potential actinomycete isolate, *Streptomyces* sp. strain NEAE-99, produces glutaminase-free L-asparaginase was isolated from a soil sample. This potential strain was identified as *S. violaceoruber* strain NEAE-99. The central composite design (CCD) approach was utilized for finding the optimal values for four variables including the mixture of soybean and wheat bran in a 1:1 ratio (w/w), the concentrations of dextrose, L-asparagine, and potassium nitrate under solid state fermentation conditions. Through the use of an artificial neural network (ANN), the production of L-asparaginase by *S. violaceoruber* has been investigated, validated, and predicted in comparison to CCD. It was found that the optimal predicted conditions for maximum L-asparaginase production (216.19 U/gds) were 8.46 g/250 mL Erlenmeyer flask of soybean and wheat bran mixture in a 1:1 ratio (w/w), 2.2 g/L of dextrose, 18.97 g/L of L-asparagine, and 1.34 g/L of KNO_3_. The experimental results (207.55 U/gds) closely approximated the theoretical values (216.19 U/gds), as evidenced by the validation. This suggests that the ANN exhibited a high degree of precision and predictive capability.

## Introduction

L-asparaginase, also known as L-asparagine amino hydrolase, is an enzyme that has anti-neoplastic activities. It is used as a potent anti-cancer therapeutic enzyme in combination with other medications in treatment protocols for various cancer types, including chronic lymphocytic leukemia (CLL), acute lymphoblastic leukemia (ALL), acute myelocytic leukemia (AML), and Hodgkin disease^1–3^.

The synthesis of L-asparagine, a necessary amino acid for the process of protein synthesis, is carried out by the enzyme L-asparagine synthetase within normal cells. Tumor cells lack L-asparagine synthetase, which prevents them from producing L-asparagine, which in turn prevents the synthesis of important proteins that rely on L-asparagine. L-asparagine is required for the growth and survival of cancer cells, and it is obtained by cancer cells through the consumption of food, absorbed, and subsequently available in the bloodstream. L-asparaginase catalyzes the hydrolysis of L-asparagine, resulting in the release of ammonia and L-aspartic acid^4^. The administration of L-asparaginase intravenously to patients with cancer reduces the concentration of L-asparagine in the bloodstream and selectively destroys tumor cells, as normal cells are capable of producing L-asparagine, which protects them from L-asparagine deficiency^5^. L-asparaginase exhibited substantial dose-dependent DPPH radical-scavenging and antioxidant activities^6^. In the food industry, when L-asparaginase is used as a catalyst for L-asparagine hydrolysis prior to frying or baking food products, the risk of hazardous acrylamide generation is greatly reduced while maintaining the original flavor appearance of the final product^7^.

L-asparaginase use in chemotherapy is restricted by various toxic effects, including neurological seizures, hyperglycemia, pancreatitis, leucopoenia, increased liver fat, hepatotoxicity, fever, skin rashes, reduced serum albumin, lipoproteins and fibrinogen and mild impairment of brain function^8,9^. However, to overcome these constraints, alternative forms of L-asparaginase have been suggested, including L-asparaginase encapsulated in erythrocytes, pegylated L-asparaginase, L-asparaginase derived from alternative sources^10^. The glutaminase activity of L-asparaginase contributes to its cytotoxicity. To prevent glutaminase-induced hypersensitivity and toxicity, it is preferable to produce L-asparaginase free of glutaminase activity^11^. Therefore, substantial research is being directed to explore a unique organism that can produce L-asparaginase without glutaminase activity. Microorganisms, particularly actinomycetes, including the *Streptomyces* species, are a potent producer of the enzyme L-asparaginase^4,12^.

Submerged fermentation (SF) has been recognized as the most common technique used for L-asparaginase production. Nevertheless, there are several disadvantages associated with this approach, including higher costs as a result of low product concentration, excessive effluent production, etc^13^. The solid-state fermentation (SSF) method is a feasible alternative to submerged fermentation and a well-established fermentation bioprocess for enzymes production. SSF includes microbial growth and metabolism on a solid moist substance containing adequate moisture to promote microbial growth where free-flowing water is limited or nearly lacking^7^. SSF is favored over submerged fermentation due to its low cost, inexpensive fermentation medium, reduced substrate inhibition and catabolite repression, higher enzyme yield, lower energy consumption, being free of organic wastewater discharge, reduced risk of contamination, and product stability^14,15^. In this study, L-asparaginase was produced under SSF bioprocess using inexpensive, readily available renewable substrates, a mixture of soybean and wheat bran.

In the field of biotechnology, statistical process optimization and artificial neural networks (ANN) are commonly used for bioprocesses optimization. ANN are machine learning algorithms within the field of artificial intelligence that develop precise and efficient models. Furthermore, it analyzes and interprets data in a manner similar to the human brain. Validation and prediction of the response were accomplished via ANN analysis of CCD results^2,16^. In comparison to CCD, ANN approach exhibited superior predictive efficacy and reduced error values^17^.

The investigation’s goals were to identify the promising *Streptomyces* sp. strain NEAE-99, which is capable of producing extracellular glutaminase-free L-asparaginase under SSF; to maximize the production of L-asparaginase by using CCD to optimize the process factors; and to compare the predictive powers of ANN and CCD models. To our best knowledge, this study is the first to demonstrate the efficacy of an AI-based optimization approach aimed to optimize process factors for maximum L-asparaginase synthesis.

## Materials and methods

### Culture conditions and microorganisms

In this investigation, *Streptomyces* sp. strain NEAE-99 was isolated from a soil sample obtained from Citrus fruit trees farm in Kafr El-Zayat city, Al Gharbiyah Governorate, Egypt. On starch nitrate agar Petri plates, *Streptomyces* sp. strain NEAE-99 was isolated. For seven days, the plates were kept in an incubator at a temperature of 30 °C. The isolate was preserved as a spore suspension in glycerol at a 20% (v/v) concentration for further investigations.

### Agar plate assay for L-asparaginase production screening

Potential actinomycetes that produced L-asparaginase were detected using the agar plates as a method of screening. The agar plates were prepared with asparagine dextrose salts (ADS) containing 0.1% K_2_HPO_4_, 1% asparagine, 0.05% MgSO_4_, 0.2% dextrose. As an indicator of pH, phenol red (0.009% in ethanol) was added to the medium. The medium’s pH was subsequently adjusted to a range of 6.8-7 prior to sterilization^18^. After being inoculated, the plates were inverted and incubated for 5–7 days at 30 °C. It was determined that the isolate with the pink zone around it produced L-asparaginase. The control plates were made with a medium without phenol red.

### Preparation of the inoculum

The spore inoculum was derived from a culture that was cultivated on a starch-nitrate agar slope for a duration of 7 days. The spores were dispersed in 10 mL of sterile production medium containing Tween 80 (0.01%, v/v) using a sterile loop^19^.

### A comparative evaluation of substrates for production of L-asparaginase under SSF

The substrate used for the enzyme production process was a mixture comprising wheat bran and soybean. In a Wiley Mill, the soybean grains were crushed to a small particle size of approximately 1–3 mm. The efficiency of soybean and wheat bran as agro-industrial waste products for L-asparaginase production was evaluated separately and in combination as a source of carbon, nutrients, and supporting material under SSF. SSF process was conducted in 250 mL Erlenmeyer flasks, utilizing an ingredient mixture consisting of 10 g of both soybean and wheat bran, with a weight-to-weight ratio of 1:1. Each Erlenmeyer flask was supplemented with freshly prepared medium (ADS broth). The contents of the Erlenmeyer flasks were thoroughly homogenized and sterilized. Two milliliters of the previously prepared inoculum were used for the inoculation. Once more, the inoculum and the Erlenmeyer flasks contents were carefully mixed applying a sterile wooden spatula. Subsequently, the flasks were incubated for a period of 5–7 days at a constant temperature of 30 °C in a stationary state.

### Preparation of the crude enzyme

Following incubation time, the crude L-asparaginase was prepared. A 90 mL solution of 0.1 M sodium phosphate buffer (pH 7) was added to each Erlenmeyer flask that contained ten grams of the fermented substrate. In a rotary shaker set at 150 rpm, the fermented substrate and sodium phosphate buffer were vigorously mixed for 1 h. A cooling centrifuge was applied to centrifuge the mixture for a duration of 15 min at 5000×g. L-asparaginase activity was assessed using the resultant supernatant as a crude enzyme.

### Assay for L-asparaginase activity

The quantification of ammonia produced by the hydrolysis of L-asparagine via direct nesslerization was used to evaluate the activity of L-asparaginase, as described by the established technique of Wriston and Yellin^20^. For the purpose of excluding the amount of ammonia produced during the microbial fermentations, one blank was made for each sample that was investigated. L-asparaginase activity was quantified in U/gds (unit/gram of fermented substrate). The quantity of L-asparaginase required to hydrolyze L-asparagine and releases one µmole of ammonia per minute at a temperature of 37 °C and 8.6 pH is known as one unit (U) of the enzyme.

### Assay of L-glutaminase

L-glutamine was utilized as the substrate to evaluate the L-glutaminase activity of the culture filtrate in accordance with the methodology established by Imada et al.^21^ .The reaction mixture is composed of 1.5 mL of 0.04 M concentration L-glutamine dissolved in 0.05 M Tris-HCl buffer (pH 8.6) and 0.5 mL of the crude enzyme. In a water bath set at 37 °C, the tubes holding the reaction mixture were shaken for thirty minutes. The reaction terminated by adding 0.5 mL of 1.5 M trichloroacetic acid to the tubes that contained the reaction mixture. The trichloroacetic acid was added first, and then the enzyme for the blank preparation. For every sample, one blank was prepared. Centrifugation was used for ten minutes at 10,000×g to separate the precipitated proteins. The quantity of the liberated ammonia was measured calorimetrically via direct nesslerization method. Nessler’s reagent (1 mL) was added to tubes containing 7 mL of distilled water and 0.5 mL of enzyme preparation. The tubes were incubated for twenty minutes at room temperature. The presence of ammonia was determined by quantifying the yellow color formation in comparison to a blank using an Optizen Pop–UV/V spectrophotometer at 450 nm. An ammonium chloride standard curve was applied to quantify the quantity of ammonia released.

### Morphological and culture characteristics

The culture characteristics like aerial spore-mass color, production of the diffusible pigments, and coloration of the substrate mycelia were examined using the methodologies outlined by Shirling and Gottlieb^22^. By culturing the isolates on Petri plates containing various ISP media including ISP medium 2–7. The inoculated Petri plates were kept in an incubator at 30 °C for a period of 14 days. In order to investigate the morphology of the spore chains and spore surfaces of *Streptomyces* sp. strain NEAE-99, scanning electron microscopy was applied in accordance with the methodology established by El-Naggar et al.^23^.

### Physiological characteristics

The formation of melanin pigments was explored on Petri plates containing either ISP media 1, 6, or 7. Furthermore, the strain’s ability to utilize a diverse array of carbon sources was evaluated using Petri plates that contained basal ISP 9 medium following the procedures that were described by Shirling and Gottlieb^22^. The following physiological features of *Streptomyces* sp. strain NEAE-99 were determined using the procedures described by El-Naggar and Abdelwahed^24^. Lecithinase activity, gelatin liquefaction, reduction of nitrates to nitrites, sodium chloride tolerance, α-amylase production, protease production, milk peptonization/coagulation, and production of cellulase. The antimicrobial activity of *Streptomyces* sp. strain NEAE-99 was evaluated against *Candida albicans*, as well as a number of Gram-negative and Gram-positive bacteria, following the procedures described by El-Naggar and Hamouda^25^. The nitrate reduction test was conducted to determine the ability of the selected isolate to reduce nitrate to nitrite using the nitrate reductase enzyme. The Griess-Ilosvay colorimetric method has been employed to detect the generated nitrite. The *Streptomyces* strain was tested for nitrate reduction using the following protocol: Test tubes containing 5 mL of nitrate broth medium (Beef extract 3 g, peptone 5 g, KNO_3_ 1 g, and Dist. H_2_O to1 L) were inoculated with a loopful of *Streptomyces* isolate spores and incubated at 28–30 °C^26^. After 3–14 days, nitrite production was detected by addition of 0.2 mL each of Griess-Ilosvay nitrite reagent solutions I and II. Tubes were shaken well and observed for development of a red colour over a period of 30 min. The intense red color was indicative of the positive reaction. Nitrite forms a compound (nitrite-sulfanilic acid) when it reacts with sulfanilic acid (reagent A). Then, nitrite-sulfanilic acid reacts with α-naphthylamine (reagent B) to give a red colour. The absence of red coloration was indicative of a negative reaction.

### 16 S rRNA extraction, sequencing, and phylogenetic analysis

The protocol of Sambrook et al.^27^ was followed to extract the DNA from the strain NEAE-99. An amplification reaction using polymerase chain reaction (PCR) was carried out in accordance with the procedure provided by El-Naggar^28^. The resultant PCR product was analyzed using agarose gel electrophoresis and the remaining mixture was purified according to the protocol described by El-Naggar^28^. The 16 S rDNA gene sequence was compared to sequences obtained from multiple databases by using the basic local alignment search tool (BLAST), which developed by Altschul et al.^29^. The MEGA-X software was used to conduct the phylogenetic analysis^30^.

### Central composite design (CCD)

The optimal values and possible interactions between a number of variables have been determined by CCD in thirty experiments. In the current study, four independent factors were investigated, including: soybean and wheat bran (1:1, w/w) (X_1_), dextrose concentration (X_2_), L-asparagine concentration (X_3_), and KNO_3_ concentration (X_4_). For each factor, five distinct levels were evaluated: – 2, – 1, 0, 1, and 2. The experiment was replicated three times. The average of the L-asparaginase activity obtained was used to determine the L-asparaginase production (Y) by *S. violaceoruber*. Design Expert 12 for Windows was chosen for carrying out both the experimental design and the statistical analysis. The CCD results were analyzed using the equation of a second-order polynomial:

1 $$Y={\beta _0}+\sum\limits_{i} {{\beta _i}{X_i}} +{\sum\limits_{{ii}} {{\beta _{ii}}{X_i}} ^2}+\sum\limits_{{ij}} {{\beta _{ij}}{X_i}{X_j}}$$

The coded level of the independent factor is symbolized by Xi, whereas the predicted L-asparaginase production is represented by Y. The symbols used to represent the coefficients of interaction, regression, quadratic, and linear terms are β_ij_, β_0_, β_ii_, and β_i_, respectively.

In order to construct three-dimensional surface graphs, STATISTICA 8 was utilized.

### Artificial neural network (ANN) analysis

The CCD analysis results were used to evaluate the influence of the four chosen process factors on L-asparaginase production by *S. violaceoruber*. JMP Pro 14 was applied for the purpose of conducting statistical analysis of the ANN. The ANN analysis comprised three layers: an input layer containing the four chosen process factors, a hidden layer, while L-asparaginase production by *S. violaceoruber* strain NEAE-99 was displayed in a single-neuron output layer. The model’s prediction accuracy was assessed on the basis of RMSE, MAD, SSE, and R^2^ values. An experimental verification was conducted to compare the predictions of the CCD and ANN models with the actual L-asparaginase production.

## Results and discussion

**Fig. 1:**
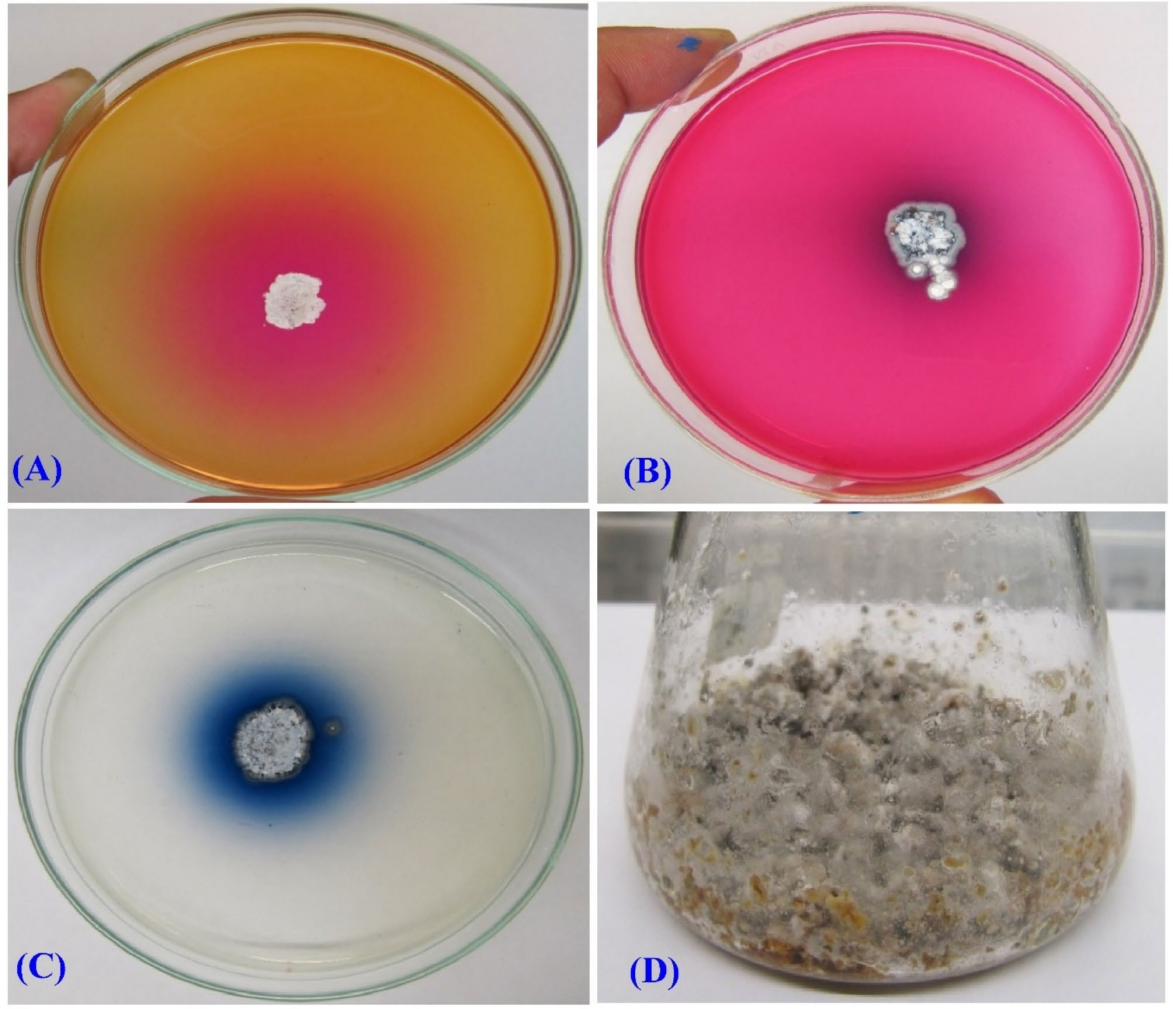
(A, B) The plate assay method used to assess L-asparaginase production after 2 and 5 days, (C) a dye-free medium prepared for the control plate, (D) *Streptomyces* sp. strain NEAE-99 growth on SSF medium.

In the next years, it is expected that the demand for L-asparaginase will expand substantially. This is due to the fact that, in addition to its applications in the medical field, it can also be used in the food processing industry^28^. Therefore, finding alternative sources of L-asparaginase could be crucial. Multiple *Streptomyces* species have been examined in terms of their L-asparaginase production, such as *S. paulus* CA01^4^, *S. olivaceus* NEAE-119^31^, alkaliphilic *S. fradiae* NEAE-82^32^. In our previous study, we collected 35 soil samples from many regions of Egypt and recovered one hundred and thirty different actinomycete strains^31^. The L-asparaginase activity of each of these isolates was evaluated using the plate assay method. As shown in Fig. 1A, B, the activity of L-asparaginase was verified through the observation of a color transition from yellow to pink in the medium that surrounds the colony. This change was in comparison with the control plate, which was prepared with medium devoid of dye (Fig. 1C).

The culture medium’s pH increased and turned pink instead of yellow due to the production of L-asparaginase by the isolate. This enzyme caused the hydrolysis of L-asparagine, resulting in the release of ammonia. The increase in pH caused by the ammonia led to the change in color of the culture medium. Among the isolates that were evaluated, *Streptomyces* sp. strain NEAE-99 emerged as a promising candidate for further research involving L-asparaginase production under SSF conditions (Fig. 1D). L-glutaminase activity was also assessed, and the findings indicated that the L-asparaginase that produced is glutaminase-free.

### Cultural and morphological characteristics of *Streptomyces* sp. strain NEAE-99

**Fig. 2:**
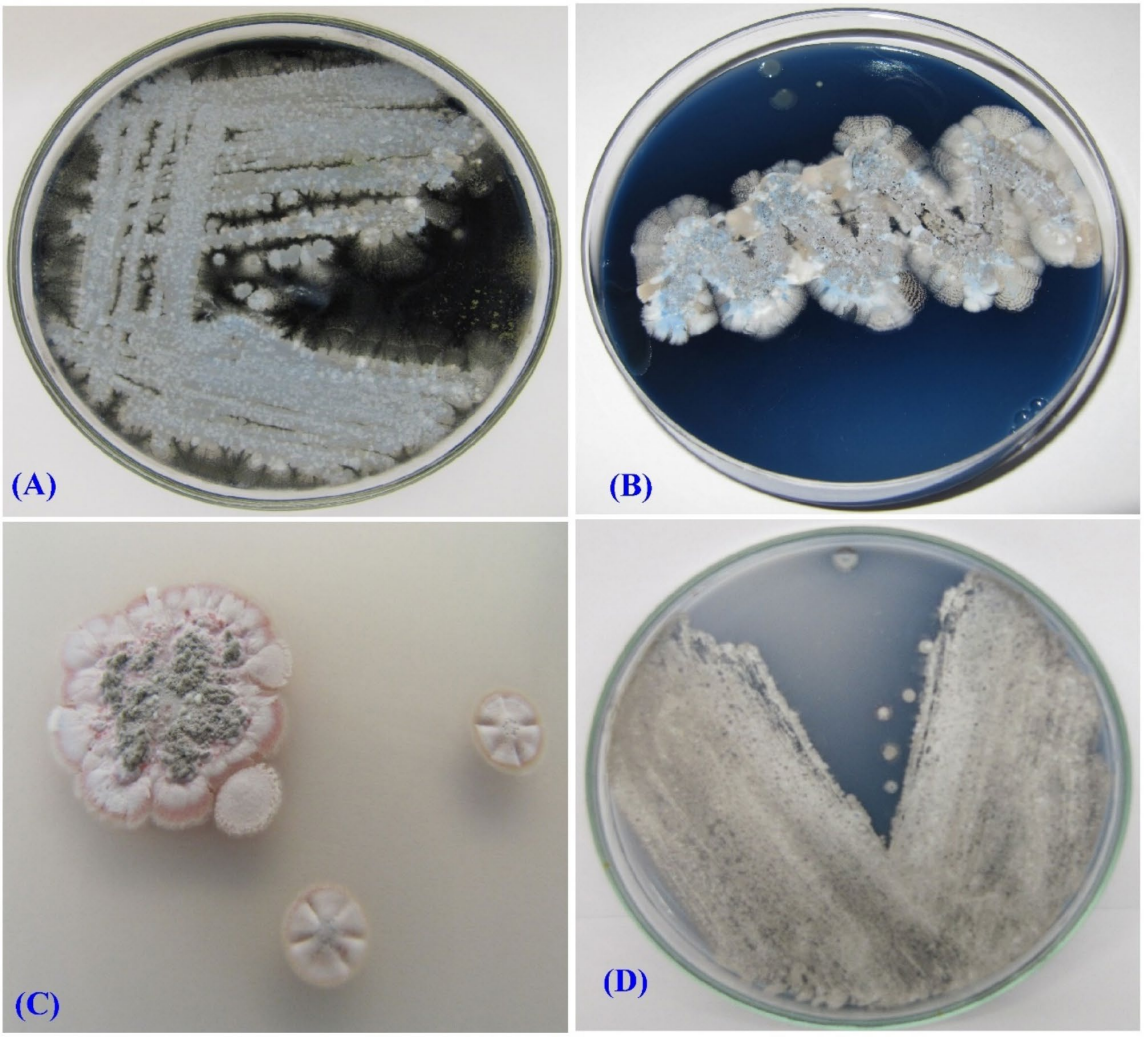
The aerial mycelium color of strain NEAE-99 upon ISP4 medium.

The culture characteristics of *Streptomyces* sp. strain NEAE-99 are displayed in Table 1. On all tested media, the strain grew well, displayed abundant, well-developed aerial mycelium that ranged in color from grey, whitish blue to bluish grey (Table 1). As shown in Table 1; Fig. 2, the aerial mycelium appeared grey on the ISP3 and ISP7 media, bluish grey color on the ISP2 and ISP4 media, and a whitish blue color on the ISP5 and ISP6 media depending on the medium components. Diffusible blue pigments are produced when the strain grown on ISP2, ISP4, ISP5 and ISP6 media. In both ISP3 and ISP7 media, violet pigment is produced.

**Table 1:** The characteristics of the culture of *Streptomyces* sp. strain NEAE-99.

Media	Overall growth	Color of the diffusible pigment	Color of the substrate mycelium	Color of the aerial mycelium.
ISP2 (Yeast extract-malt extract agar)	Excellent	Blue	Dark blue	Bluish grey
ISP3 (oatmeal agar)	Good	Violet	Violet	Grey
ISP4 (inorganic salt-starch agar)	Excellent	Blue	Dark blue	Bluish grey
ISP5 (glycerol asparagine agar)	Excellent	Blue	Blue	Whitish blue
ISP6 (peptone-yeast extract iron agar)	Good	Blue	Blue	Whitish blue
ISP7 (tyrosine agar)	Excellent	Violet	Violet	Grey

**Fig. 3:**
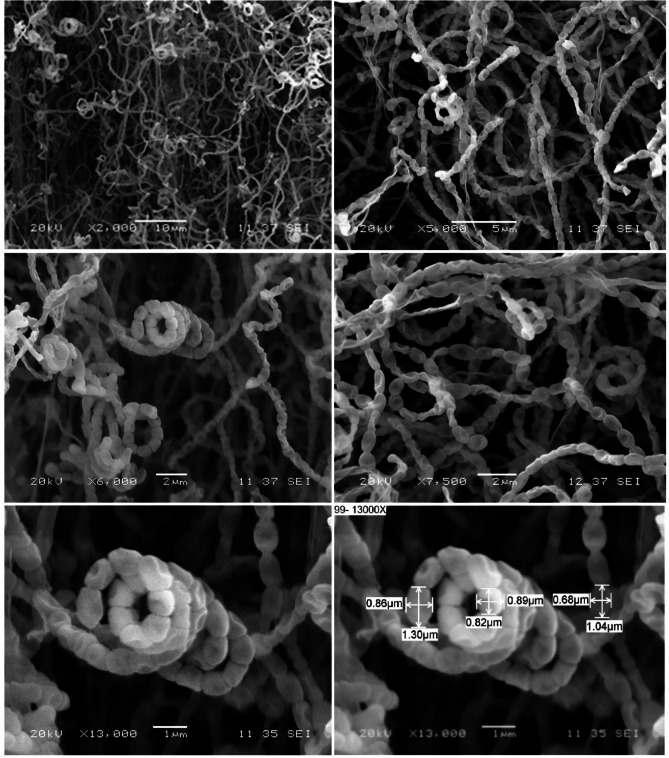
SEM showing the spore surface and morphological characteristics of the spore-chains of strain NEAE-99 at magnifications ranging from 2000 to 13,000 ×.

Using a scanning electron microscope (SEM) at magnifications ranging from 2000× to 13,000×, the morphology of *Streptomyces* sp. strain NEAE-99 cultured on ISP2 medium was examined. A scanning electron micrograph indicates the absence of verticils and mycelium fragmentation. The aerial mycelium differentiated into long spiral-shaped spore chains, which may be closed or opened. More than fifty elongated, smooth-surfaced spores with diameters ranging from 0.68 to 0.86 × 0.89 to 1.30 μm can be observed in spore chains (Fig. 3). The morphological characteristics of strain NEAE-99 align with those recognized among the *Streptomyces* genus^33^.

### Physiological properties of strain NEAE-99

The physiological characteristics of the strain NEAE-99 are displayed in Table 2. The strain grew at pH values of 5, 7, and 9 and a temperature ranging from 25 to 40 °C. It was determined that the optimal temperature and pH were 30 °C and 7, respectively. The formation of melanoid pigments was not observed. The strain exhibited a moderate resistance to NaCl, with a maximum concentration of 4% (w/v). The strain was grown using the following sugars: Trehalose, D (–) fructose, D (+) mannose, D (+) glucose, rhamnose, maltose, cellulose, L-arabinose, ribose, and D (+) galactose. *Streptomyces* sp. strain NEAE-99 produced α –amylase, L-asparaginase, protease, cellulase, uricase, chitosanase, lecithinase and gelatinase. *Streptomyces* sp. strain NEAE-99 has the capability to degrade lecithin, casein, and starch. *Streptomyces* sp. strain NEAE-99 displayed positive coagulation and peptonization of milk, but it showed a negative reduction of nitrate to nitrite (Table 2). No antibacterial activity has been seen against any of the tested bacterial or fungal strains.

**Table 2:** *Streptomyces* sp. strain NEAE-99 physiological characteristics.

The properties	*Streptomyces* sp. strain NEAE-99
Growth temperature range	25–40 °C
Growth in pH	5, 7, 9
Production of melanin in ISP 1, ISP 6, and ISP 7 media	−
Maximum concentration of NaCl resistance	4%, w/v
Growth on sugars (1%, w/v)	
D (+) Galactose, D (-) Fructose, Maltose, Rhamnose, Cellulose, Trehalose, D (+) Glucose	+
Ribose, L-arabinose, D (+) Mannose	±
Enzymes production: α –amylase, L-asparaginase, protease, cellulase, uricase, chitosanase, lecithinase and gelatinase	+
Degradation of lecithin, casein and starch	+
Nitrate reduction to nitrite	−
Milk coagulation and peptonization	+
Antimicrobial activities against*Rhizoctonia solani*, *Candida albicans*, *Alternaria solani*, *Sacchromyces cerevisiae*, *Aspergillus niger*, *Fusarium oxysporum*, *Bipolaris oryzae*, *Escherichia coli*, *Klebsiella* sp., *Pseudomonas aeruginosa*, *Bacillus subtilis*, *Staphylococcus aureus*	−

–, Negative ; +, Positive ; ±, Doubtful.

### Phylogenetic analysis

**Fig. 4:**
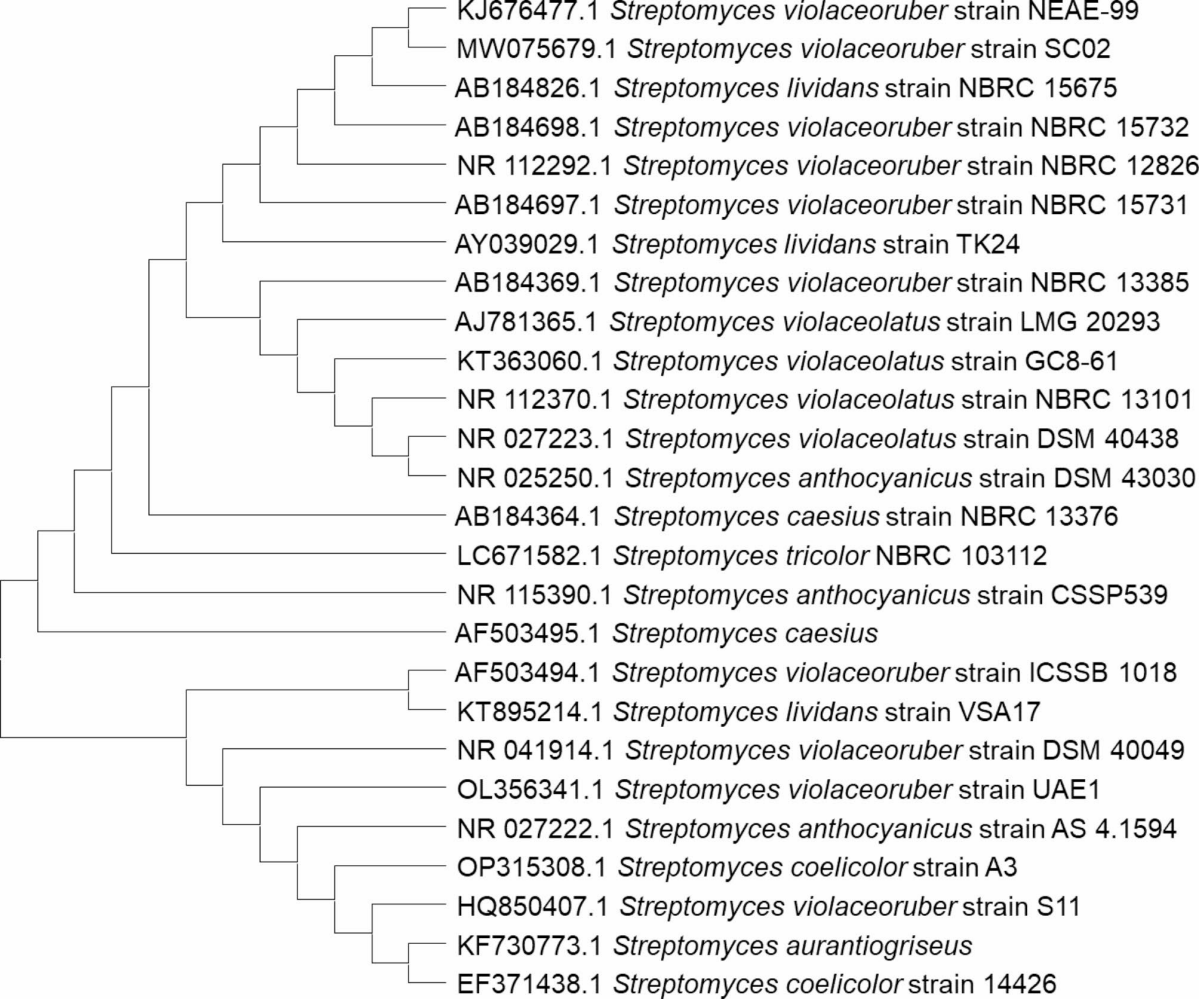
A neighbor-joining phylogenetic tree demonstrates the evolutionary relationship between similar *Streptomyces* species and *Streptomyces* sp. strain NEAE-99.

The 16S rRNA gene sequence for strain NEAE-99 has been determined to be 1537 base pairs in length. The DNA sequencing data has been formally submitted to the GenBank database and assigned the accession number KJ676777. A substantial similarity between the strain under study and numerous species of the *Streptomyces* genus was noted via a GenBank database search using BLAST^29^. The neighbor-joining algorithm technique was applied in order to create a phylogenetic tree^34^ and the MEGA-X software^30^ using the 16 S rDNA gene sequences of members of the *Streptomyces* genus (Fig. 4).

*Streptomyces* sp. strain NEAE-99 exhibits a close phylogenetic relationship to certain related *Streptomyces* species, as illustrated by the phylogenetic tree. According to the phylogenetic tree, *Streptomyces* sp. strain NEAE-99 is a member of the same clade with *S. violaceoruber* strain SC02 (Accession No. MW075679.1), with an identity percentage of 99.93%. *Streptomyces* sp. strain NEAE-99 is closely related to the type strain of *S. violaceoruber* owing to its physiological, morphological, and culture characteristics. Consequently, *Streptomyces* sp. strain NEAE-99 has been identified as *S. violaceoruber* strain NEAE-99.

### Optimization of L-asparaginase production by *S. violaceoruber* strain NEAE-99 using central composite design

The process of SSF is being optimized with the goal of enhancing L-asparaginase production through the use of ingredients that are less expensive in order to minimize the costs of production. Selecting an appropriate solid substrate is one of the most critical steps in SSF. The appropriateness of some agro-industrial wastes, like soybean and wheat bran (either separately or in combination), was assessed in our earlier study by El-Naggar et al.^35^ as carbon sources and as supporting material that has been loaded with all the nutrients required for *S. violaceoruber* growth and L-asparaginase production. The results demonstrate that two substrates significantly increased *S. violaceoruber*’s production of L-asparaginase.: wheat bran (18.088 U/gds) and soybean (27.985 U/gds). Nevertheless, the highest L-asparaginase production by *S. violaceoruber* during SSF was achieved by using a mixture of soybean and wheat bran in a 1:1 ratio by weight, resulting in a yield of 41.864 U/gds (Fig. 1D). As the mixture of soybean and wheat bran, in a 1:1 ratio by weight, yielded the highest L-asparaginase production, so it was chosen for more fermentation studies to produce L-asparaginase under SSF. Because soybeans contain significant amounts of minerals, carbohydrates, lipids, and proteins, they are a suitable substrate for L-asparaginase production^35^. According to a study conducted by Isaac and Abu-Tahon^36^, *Fusarium solani* AUMC 8615 achieved the highest activity of L-asparaginase (187.9 U/mL) on wheat bran, the most favorable natural substrate out of the seven substrates studied.

The CCD design matrix comprises 30 experimental trials with 6 replicates at the midpoints (5, 6, 7, 10, 17, and 24) used in order to achieve the highest possible L-asparaginase production and to investigate the linear, quadratic, and mutual interactions between four independent variables including: soybean and wheat bran (1:1, w/w) (X_1_), concentration of dextrose (X_2_), concentration of L-asparagine (X_3_), and concentration of KNO_3_ (X_4_) (Table 3).

**Table 3:** Central composite design for L-asparaginase production by *S. violaceoruber* strain NEAE-99.

Std	Run	X_1_	X_2_	X_3_	X_4_	L-asparaginase production (U/gds)					
						Experimental results	CCD		ANN		
							Predicted results	Residuals	Predicted results	Residuals	Validation
9	1	− 1	− 1	− 1	1	27.23	15.49	11.75	28.37	− 1.14	Training
23	2	0	0	0	− 2	24.17	25.40	− 1.23	31.16	− 6.99	Training
17	3	− 2	0	0	0	73.13	69.96	3.17	73.05	0.08	Training
20	4	0	2	0	0	150.03	150.02	0.01	149.53	0.50	Training
27	5	0	0	0	0	172.17	175.60	− 3.43	175.43	− 3.26	Training
25	6	0	0	0	0	170.59	175.60	− 5.01	175.43	− 4.84	Training
26	7	0	0	0	0	171.12	175.60	− 4.48	175.43	− 4.31	Training
2	8	1	− 1	− 1	− 1	110.14	106.21	3.93	109.72	0.42	Training
3	9	− 1	1	− 1	− 1	91.88	90.36	1.52	91.89	− 0.01	Training
29	10	0	0	0	0	191.33	175.60	15.73	175.43	15.90	Training
19	11	0	− 2	0	0	90.14	95.02	− 4.88	85.92	4.22	Validation
6	12	1	− 1	1	− 1	38.64	30.72	7.91	41.77	− 3.13	Training
5	13	− 1	− 1	1	− 1	17.89	17.83	0.06	20.26	− 2.37	Training
1	14	− 1	− 1	− 1	− 1	69.52	75.31	− 5.79	70.43	− 0.91	Validation
15	15	− 1	1	1	1	126.38	124.87	1.51	132.50	− 6.12	Training
14	16	1	− 1	1	1	99.79	95.87	3.92	103.89	− 4.10	Validation
28	17	0	0	0	0	179.15	175.60	3.55	175.43	3.72	Training
22	18	0	0	2	0	60.40	63.11	− 2.71	51.94	8.46	Training
13	19	− 1	− 1	1	1	82.06	86.36	− 4.30	88.88	− 6.82	Training
7	20	− 1	1	1	− 1	63.35	61.07	2.28	64.57	− 1.22	Training
10	21	1	− 1	− 1	1	40.15	42.99	− 2.84	39.55	0.60	Validation
18	22	2	0	0	0	103.77	111.80	− 8.04	105.63	− 1.86	Training
24	23	0	0	0	2	22.34	25.98	− 3.64	24.04	− 1.70	Training
30	24	0	0	0	0	169.24	175.60	− 6.36	175.43	− 6.19	Training
11	25	− 1	1	− 1	1	17.32	25.80	− 8.48	21.81	− 4.49	Training
21	26	0	0	− 2	0	37.36	39.52	− 2.16	28.06	9.30	Training
8	27	1	1	1	− 1	69.10	75.41	− 6.31	67.18	1.92	Validation
4	28	1	1	− 1	− 1	126.44	122.70	3.74	127.49	− 1.05	Training
12	29	1	1	− 1	1	60.13	54.75	5.38	62.19	− 2.06	Training
16	30	1	1	1	1	141.05	135.82	5.23	141.88	− 0.83	Validation

Variable	Code	− 2	− 1	0	1	2
Soybean and wheat bran (g/250 mL Erlenmeyer flask)	X_1_	5	10	15	20	25
Dextrose (g/L)	X_2_	0	1	2	3	4
L-asparagine (g/L)	X_3_	0	5	10	15	20
KNO_3_ (g/L)	X_4_	0	0.5	1	1.5	2

In addition, Table 3 presents the experimental and theoretical findings of L-asparaginase production for a variety of combinations of the four independent factors. The results indicated that the production of L-asparaginase was significantly influenced by the concentrations of four independent factors. The greatest yield of L-asparaginase, 191.33 U/gds, was achieved in central run number 10 using the optimal experimental conditions: 15 g/250 mL Erlenmeyer flask of a mixture consisting of equal weight ratios of soybean and wheat bran, supplemented with freshly prepared ADS broth containing (g/L): L-asparagine 10, dextrose 2, and KNO_3_. (1) Additionally, run number 23 yielded the minimal production of L-asparaginase, measuring 22.34 U/gds. This occurred when the fermentation medium consisted of 15 g/250 mL Erlenmeyer flask of a mixture consisting of equal weight ratios of soybean and wheat bran, supplemented with freshly prepared ADS broth containing (g/L): of dextrose 2, L-asparagine 10, and of KNO_3_. (2) The decrease in L-asparaginase production during run no. 23 may have been caused by an elevated concentration of KNO_3_.

### Multiple regression analysis and ANOVA

Tables 4, and 5 present the results of the multiple regression analysis and ANOVA performed to investigate the extracellular L-asparaginase production using *S. violaceoruber* as influenced by the effects of the independent process factors namely: soybean and wheat bran (1:1, w/w) (X_1_), concentration of dextrose (X_2_), concentration of L-asparagine (X_3_), and concentration of KNO_3_ (X_4_). An assessment of the model’s validity was conducted by analyzing the data provided in Table 4, including the coefficient estimates and the values of determination coefficient (R^2^), predicted R^2^, adjusted R^2^, *F*-value (Fisher value), *P*- (probability value). Furthermore, an assessment of lack of fit was conducted to verify the precision of the model. The model used exhibited an R^2^ value of 0.9891 (Table 4), indicates that the model accurately predicts 98.91% of the variation in L-asparaginase production, with only 1.09% of the whole variation remaining unexplained. R^2^ values, ranging from 0 to 1, indicate the degree to which variability in measured response values may be attributed to the variables used in the experiment and their interactions. A greater R^2^ value (approximate 1) signifies a more robust model and a higher capacity for predicting the response^2^. The high R^2^ value indicates a robust correlation between the predicted and measured response values, revealing the variability of the response values around their mean.

**Table 4:** ANOVA for extracellular L-asparaginase production using *S. violaceoruber* as influenced by the process factors.

Source of variance		Coefficient estimate	Sum of Squares	Df	Mean Square	F-value	*P*-value
Model	Intercept	175.60	87514.49	14	6251.04	97.07	< 0.0001*
Linear effect	X_1_	10.46	2626.78	1	2626.78	40.79	< 0.0001*
	X_2_	13.75	4537.75	1	4537.75	70.46	< 0.0001*
	X_3_	5.90	834.56	1	834.56	12.96	0.0026*
	X_4_	0.15	0.51	1	0.51	0.01	0.9303
Interaction effect	X_1_ X_2_	0.36	2.09	1	2.09	0.03	0.8593
	X_1_ X_3_	− 4.50	324.08	1	324.08	5.03	0.0404*
	X_1_ X_4_	− 0.85	11.46	1	11.46	0.18	0.6791
	X_2_ X_3_	7.05	794.94	1	794.94	12.34	0.0031*
	X_2_ X_4_	− 1.18	22.44	1	22.44	0.35	0.5638
	X_3_X_4_	32.09	16476.87	1	16476.87	255.86	< 0.0001*
Quadratic effect	X_1_²	− 21.18	12304.48	1	12304.48	191.07	< 0.0001*
	X_2_²	− 13.27	4830.21	1	4830.21	75.01	< 0.0001*
	X_3_²	− 31.07	26481.14	1	26481.14	411.21	< 0.0001*
	X_4_²	− 37.48	38525.82	1	38525.82	598.24	< 0.0001*
Error effect	Lack of Fit		608.54	10	60.85	0.85	0.6141
	Pure Error		357.43	5	71.49		
R^**2**^	0.9891	Std. Dev.	8.02				
Adj R^**2**^	0.9789	Mean	93.2				
Pred R^**2**^	0.9546	C.V. %	8.61				
Adeq Precision	28.22	PRESS	4019.9				

*: Significant values, *F*: Fishers’s function, *Df*: degree of freedom, *P*: Level of significance.

The adjusted-R^2^ value of the regression model used for studying the production of L-asparaginase by *S. violaceoruber* was determined to be 0.9789. A higher adjusted R^2^ value implies a high level of model accuracy. The adjusted coefficient of determination (adjusted-R^2^) provides an explanation for the variation in the response as influenced by the independent factors. The current model’s predicted-R^2^ value of 0.9546 indicates its significance for predicting L-asparaginase production in future experiments. The theoretical and experimental values of L-asparaginase production are significantly correlated, as evidenced by the high level of agreement among the predicted-R^2^ value of 0.9546 and the adjusted-R^2^ value of 0.9789. When evaluating the predictive power of the model to predict the response values at various levels of the assessed process variables in the future experiments, the predicted R^2^ is applied^37^.

Interactions between two variables can be categorized into two categories: Antagonism is demonstrated by negative coefficient values, which suggest an antagonistic relationship among the variables, and synergism, indicated by a positive coefficient that means a synergistic interaction between the variables. Table 4 clearly indicates that the coefficients values of the four process factors are positive. These variables included soybean and wheat bran (1:1, w/w) (X_1_), concentration of dextrose (X_2_), L-asparagine concentration (X_3_), and KNO_3_ concentration (X_4_). These positive coefficients values indicate that the process factors increase L-asparaginase production using *S. violaceoruber* within the ranges that were evaluated. Furthermore, the mutual interaction effects between X_1_ and X_2_; X_2_ and X_3_; X_3_ and X_4_ had a positive influence on L-asparaginase production by *S. violaceoruber*. The data indicated that the mutual interaction effects between X_3_ and X_4_, as well as X_2_ and X_3_, have significant positive coefficients with higher coefficient estimates (32.09 and 7.05, respectively) and lower *P*-values (< 0.0001 and 0.0031, respectively). This meaning that they act as limiting factors, and their mutual interaction effects will enhance L-asparaginase production by *S. violaceoruber*. Conversely, the coefficient estimates of 0.36 indicates that the mutual interaction effects between X_1_ and X_2_ are negligible positive coefficients with the lowest effect. On the other hand, the quadratic impacts of all process variables and the mutual interaction impacts between X_1_ and X_3_; X_1_ and X_4_; X_2_ and X_4_ have negative coefficient values (Table 4), which indicates that they have a negative impact on L-asparaginase production by *S. violaceoruber*. It can be concluded that the variable significantly influences the response when the value of the calculated coefficients is large, irrespective of their sign. If the coefficient value is close to zero, it is hypothesized that the variable has minimal or no impact on the production. If the coefficient is positive, it suggests that production increases as the value of the tested variable increases. In contrast, a negative sign signifies an increase in production when the variable reaches its minimum value. The variable exerts a negligible or nonexistent influence on the ultimate outcome, as indicated by a coefficient value approaching zero. A positive coefficient associates with an increase in the value of the tested variable, signifying that such an increase corresponds to a rise in production. Conversely, when the variable reaches its minimum value, a negative sign signifies an increase in production.

The significance of individual variables is evaluated, and interactions among the factors under consideration is determined using *P-*values and *F*-values. Moreover, it was established that process factors are assumed to significantly affect the response if their *P*-values are < 0.05^37^. The regression model’s significance is demonstrated by the *F*-value of 97.07 and the *P*-value of less than 0.0001. L-asparaginase production by *S. violaceoruber* is significantly impacted by the linear effects of X_1_, X_2_, and X_3_, as indicated by *P*-values less than 0.05 and *F*-values of 40.79, 70.46 and 12.96; respectively (Table 4). On the basis of the *F*-value of 0.01 and the probability value of 0.9303, it is evident that the linear effect of KNO_3_ (X_4_) has no significant effect on the production of L-asparaginase by *S. violaceoruber* (Table 4). Conversely, *P*-value < 0.0001 and a negative coefficient estimate value of -37.48 support the importance of the quadratic impact of KNO_3_, indicates that it greatly decreases the production of L-asparaginase. The mutual interaction effects between X_1_ X_3_ (*P*-values of 0.0404); X_2_ X_3 _(*P*-values of 0.0031) and X_3_X_4_ (*P*-values of < 0.0001) have significant effects on the production of L-asparaginase by *S. violaceoruber*. Conversely, the *P*-values of 0.8593, 0.6791, and 0.5638 indicate that the mutual interactions effects between X_1_ X_2_; X_1_ X_4_ and X_2_ X_4_; respectively, do not exert statistically significant impacts on production of L-asparaginase by *S. violaceoruber*. The significance of the quadratic impacts of X_1_, X_2_, X_3_, and X_4_ is supported by *P*-values below 0.0001, which indicate that these impacts have a substantial influence on L-asparaginase production. The C.V. %, mean, Std. Dev. and PRESS values are 8.61, 93.2, 8.02 and 4019.9; respectively. When evaluating the signal-to-noise ratio, the value of adequate precision is crucial, the adequate precision value in the current study is 28.22. The precision of the model can be determined by a signal-to-noise ratio that is more than 4 which is considered to be desirable^38^.

The following equation can be used to determine the maximal predicted production of L-asparaginase (Y):

2 $$\begin{aligned} {\text{Y}} & \,=\,{\text{175}}.{\text{6}}0\,+\,{\text{1}}0.{\text{46}}{{\text{X}}_{\text{1}}}\, - \,{\text{13}}.{\text{75}}{{\text{X}}_{\text{2}}}\,+\,{\text{5}}.{\text{9}}0{{\text{X}}_{\text{3}}}\,+\,0.{\text{15}}{{\text{X}}_{\text{4}}}\, \\ & \;\;+\,0.{\text{36}}{{\text{X}}_{\text{1}}}{{\text{X}}_{\text{2}}}\, - \,{\text{4}}.{\text{5}}0{{\text{X}}_{\text{1}}}{{\text{X}}_{\text{3}}}\, - \,0.{\text{85}}{{\text{X}}_{\text{1}}}{{\text{X}}_{\text{4}}}\,+\,{\text{7}}0.0{\text{5}}{{\text{X}}_{\text{2}}}{{\text{X}}_{\text{3}}}\, - \,{\text{1}}.{\text{18}}{{\text{X}}_{\text{2}}}{{\text{X}}_{\text{4}}}\, \\ & \;\;+\,{\text{32}}0.0{\text{9}}{{\text{X}}_{\text{3}}}{{\text{X}}_{\text{4}}}\, - \,{\text{21}}.{\text{18}}{{\text{X}}^{\text{2}}}_{{\text{1}}}\, - \,{\text{13}}.{\text{27}}{{\text{X}}^{\text{2}}}_{{\text{2}}}\, - \,{\text{31}}0.0{\text{7}}{{\text{X}}^{\text{2}}}_{{\text{3}}}\, - \,{\text{37}}.{\text{48}}{{\text{X}}^{\text{2}}}_{{\text{4}}} \\ \end{aligned}$$

X_1_, X_2_, X_3_, and X_4_ are the values of the independent factors including soybean and wheat bran (1:1, w/w) (X_1_), concentration of dextrose (X_2_), concentration of L-asparagine (X_3_), and concentration of KNO_3_ (X_4_).

An appropriate and highly significant polynomial model is chosen from linear, 2FI, and quadratic models that suit L-asparaginase production by *S. violaceoruber*, depending on the results of the fit summary in Table 5. The results indicate that the quadratic model has an insignificant lack of fit (*P*-value = 0.6141, *F*-value = 0.85) and a very small *P*-value (< 0.0001). Consequently, the model is considered appropriate model and highly significant for L-asparaginase production by *S. violaceoruber* (Table 5). In addition, the quadratic model exhibits greater efficiency compared to other models, as shown by its higher predicted R^2^ value (0.9546), adjusted R^2^ value (0.9789), and R^2^ value (0.9891). In addition, the quadratic model’s summary statistics indicated a lower PRESS value of 4019.9 and a lower standard deviation of 8.02 which demonstrate the validity of the model and its capacity to represent the data accurately.

**Table 5:** Fit summary results for CCD.

Model summary statistics					
Source	Std. Dev.	*R*²	Adjusted *R*²	Predicted *R*²	PRESS
Linear	56.74	0.0904	-0.0551	-0.1926	1.06E + 05
2FI	57.51	0.2897	-0.0842	-0.1718	1.04E + 05
Quadratic	8.02	0.9891	0.9789	0.9546	4019.9

Source	Sum of squares	Df	Mean square	F-value	*P*-value
Sequential model sum of squares					
Linear vs. Mean	7999.59	4	1999.90	0.62	0.6516
2FI vs. Linear	17631.89	6	2938.65	0.89	0.5226
Quadratic vs. 2FI	61883.01	4	15470.75	240.24	< 0.0001*
Lack of fit tests					
Linear	80123.44	20	4006.17	56.04	0.0001*
2FI	62491.56	14	4463.68	62.44	0.0001*
Quadratic	608.54	10	60.85	0.85	0.6141

*P*-value: Level of significance, *F*-value: Fishers’s function,, 2FI: two factors interaction, *Df*: degree of freedom, *: Significant values.

### Three-dimensional (3D) surface plots

**Fig. 5:**
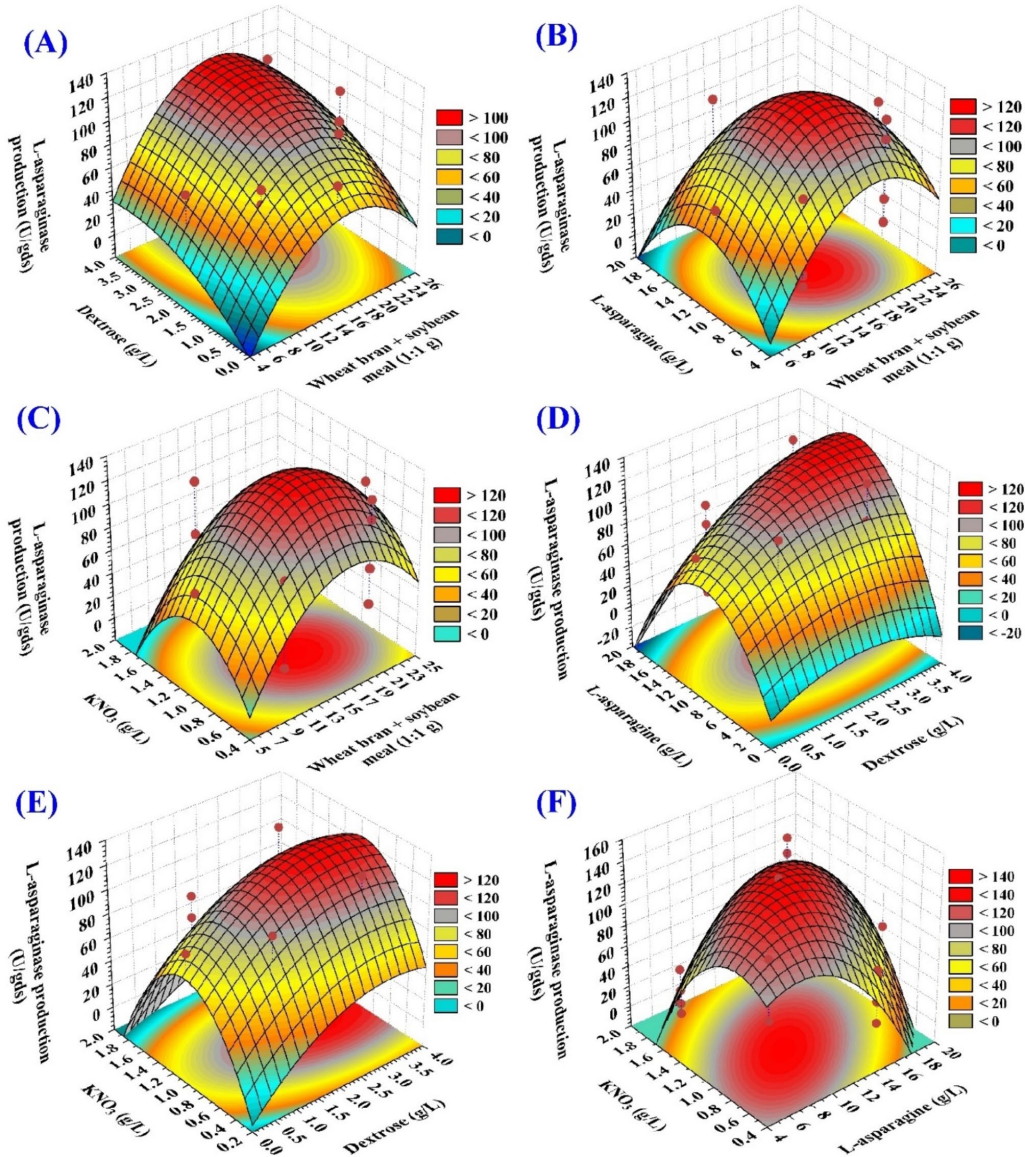
3D plots demonstrate L-asparaginase production using *S. violaceoruber* as influenced by four process factors, their interactions, and their optimal concentrations required for achieving the highest production of L-asparaginase.

To elucidate the impact of the variables being investigated, their interactions, and the optimal concentrations needed to achieve maximum L-asparaginase production, 3D surface plots have been created. Figure 5A–C illustrates L-asparaginase production on the Z-axis as a function of a pair of variables, while keeping the values of the other two variables at their zero levels.

Figure 5A demonstrates the effects of soybean and wheat bran mixture (g/250 mL Erlenmeyer flask) (X_1_), dextrose concentration (X_2_) on the L-asparaginase production using *S. violaceoruber*, while maintaining L-asparagine (X_3_) and KNO_3_ (X_4_) at their zero levels. The experimental results demonstrate that at low as well as high levels of soybean and wheat bran (g/250 mL Erlenmeyer flask) and dextrose, production of L-asparaginase was decreased. The highest yield of L-asparaginase was achieved at moderate levels of both soybean and wheat bran mixture (g/250 mL Erlenmeyer flask) and dextrose concentration. Consequently, the point prediction tool of Design expert software (version 12) was applied to determine the optimal value for each factor in order to achieve the highest predicted production of L-asparaginase. It is possible to achieve the maximum production of 180.47 U/gds L-asparaginase by employing a mixture of 16.4 g/250 mL Erlenmeyer flask of soybean and wheat bran in a ratio of 1:1; w/w, dextrose (2.5 g/L), when the concentrations of L-asparagine and KNO_3_ maintained at their central levels (at 10, 1 g/L; respectively). The results of this study agree with the findings of El-Naggar et al.^35^ , who reported that *S. brollosae* NEAE-115 utilized soybean and wheat bran in a ratio of 1:1; w/w as substrates for L-asparaginase production under SSF conditions. Various investigations on enzyme production under SSF conditions have shown that, the use of numerous substrates increases the production of metabolites and microbial growth more than the use of a single substrate alone^39,40 ,41^. According to Sharma and Mishra^42^, It has been shown that combining a variety of substrates can provide a wide range of nutrients that are not available from a single source. By combining soybean, wheat bran, and other inexpensive substrates, a significant number of nutrients can be obtained. Dharmsthiti and Luechai^43^ discovered that *Aspergillus niger* AK10 effectively used soybean as a substrate under SSF conditions. Soybean is an ideal for L-asparaginase production due to its high content of minerals, lipids, proteins, and carbohydrates. Dextrose (glucose) has been found to be the most effective carbon source for promoting the production of L-asparaginase by *S. olivaceus* NEAE-119 during submerged fermentation at a concentration of 3 g/L^44^. The utilization of cost-effective agricultural byproducts, such as rice bran, sesame oil cake, wheat bran, soybean meal, groundnut oil cake, and tea trash, aids in cost reduction and supports environmental sustainability^45,46^.

Figure 5B illustrates how different amounts of soybean and wheat bran (g/250 mL Erlenmeyer flask) (X_1_), L-asparagine concentration (X_3_) affect L-asparaginase production, while maintaining concentrations of both dextrose (X_2_) and KNO_3_ (X_4_) at their zero levels. The experimental results demonstrate that at low as well as higher levels of both L-asparagine and soybean and wheat bran (g/250 mL Erlenmeyer flask), L-asparaginase production was decreased. Abdel-Hamid et al.^47^. reported that a significant decrease in L-asparaginase production was observed when the concentration of L-asparagine was increased. This reduction may be attributed to the negative impact of L-asparagine on L-asparaginase gene expressions or the downregulation of nitrogenous compound availability. On the other hand, the reduction in L-asparaginase production at elevated concentrations of soybean and wheat bran may be attributed to catabolic inhibition effect or enzyme inactivation. It was reported that the production of microbial L-asparaginase faces catabolic inhibition and required less amount of carbon source^48^. A maximum yield of L-asparaginase was achieved at moderate levels of both soybean and wheat bran mixture (g/250 mL Erlenmeyer flask) and L-asparagine concentration. It is possible to achieve the highest predicted L-asparaginase production of 177.041 U/gds by using 16.4 g/250 mL Erlenmeyer flask from soybean and wheat bran mixture and 10.40 g/L of L-asparagine, while the concentrations of dextrose and KNO_3_ were maintained at their respective central levels (at 2, 1 g/L; respectively). The results of the study indicate that the production of L-asparaginase is significantly impacted by the nitrogen source. Microorganisms utilize various organic and inorganic nitrogen sources in order to produce vital biological substances, such as industrial enzymes, proteins, nucleic acids, and amino acids, as well as the cell wall^49^.

Figure 5C illustrates the effects of soybean and wheat bran (X_1_), KNO_3_ (X_4_) on L-asparaginase production, while keeping dextrose (X_2_) and L-asparagine (X_3_), at their zero levels. In Fig. 5C, it was seen that the maximum amount of L-asparaginase was produced at intermediate concentrations of both soybean and wheat bran mixture and KNO_3_. Increased levels of these variables have been shown to reduce L-asparaginase production. It is possible to achieve the highest predicted L-asparaginase production of 176.88 U/gds by using 16.2 g/250 mL Erlenmeyer flask from the mixture of soybean and wheat bran and 1.01 g/L of KNO_3_, while the concentrations of dextrose and L-asparagine were maintained at their respective central levels (at 2, 10 g/L; respectively). The increase in L-asparaginase production resulting from the addition of KNO_3_ suggests that nitrogen is involved in the regulation of its production. For the production of L-asparaginase by *B. licheniformis*, ammonium sulfate was found to be the most efficient nitrogen source (the enzyme production increased by 35.56%) when compared to other nitrogen sources including asparagine, casein, ammonium chloride, sodium nitrate, yeast extract, urea and potassium nitrate^50^.

Figure 5D illustrates the impacts of dextrose (X_2_) and L-asparagine (X_3_) on L-asparaginase production, while maintaining the level of soybean and wheat bran mixture (X_1_) and KNO_3_ (X_4_) at their zero levels. Figure 5D demonstrated that L-asparaginase production increase with the increase in the concentration of both dextrose (X_2_) and L-asparagine (X_3_). At high concentrations of both dextrose (X_2_) and L-asparagine (X_3_), L-asparaginase production decreased. The highest L-asparaginase production is observed when the levels of dextrose (X_2_) and L-asparagine (X_3_) are beyond middle levels. L-asparaginase production is enhanced when carbon concentrations are increased, yielding considerable quantities of the enzyme^51^. It has been observed that the higher concentrations of dextrose (glucose) act as a catabolic repressor in the bacterial production of L-asparaginase by *Erwinia aeroideae* and *Escherichia coli*^52,53^. This may be attributed to the suppression of catabolites of lactate transport components, which promoted the production of L-asparaginase^54^. The highest predicted L-asparaginase production of 179.87 U/gds can be achieved by applying 2.5 g/L dextrose and 10.8 g/L of L-asparagine, while the concentrations of soybean and wheat bran mixture (X_1_) and KNO_3_ concentration (X_4_) were maintained at their respective central levels (at 15 g/250 mL Erlenmeyer flask, 1 g/L; respectively).

Figure 5E shows the effects of dextrose (X_2_) and KNO_3_ (X_4_) on production of L-asparaginase, while keeping soybean and wheat bran mixture (X_1_) and L-asparagine (X_3_) at their zero levels. Figure 5E demonstrated that the production of L-asparaginase was induced by an increase in the concentrations of dextrose (X_2_) and KNO_3_ (X_4_), and then the production of L-asparaginase was subsequently reduced by a subsequent increase in the concentrations may be due to catabolic inhibition effect. The highest predicted L-asparaginase production of 179.13 U/gds can be achieved by applying 2.5 g/L dextrose and 1.01 g/L of KNO_3_, while the concentrations of soybean and wheat bran mixture, and L-asparagine were maintained at their central levels (at 15 and 10 g/L; respectively).

Figure 5F displays the effects of L-asparagine (X_3_) and KNO_3_ (X_4_) on production of L-asparaginase, while maintaining soybean and wheat bran mixture (X_1_), and dextrose (X_2_), at their zero levels. A subsequent increase in the concentrations of L-asparagine (X_3_) and KNO_3_ (X_4_) to their moderate levels resulted in an increase in the production of L-asparaginase. In general, the utilization of various nitrogen sources can be utilized to enhance enzyme production. A further increase in their concentration resulted in a decrease in the production of L-asparaginase. The decrease in L-asparaginase production at higher concentrations may be attributed to the nitrogen regulation of L-asparaginase production at excess L-asparagine and KNO_3_, which may be a result of the catabolic inhibition effect. It is possible to achieve the highest predicted L-asparaginase production of 175.95 U/gds by using 10.5 g/L L-asparagine and 1.02 g/L of KNO_3_, while the concentrations of soybean and wheat bran, and dextrose maintained at their central levels (at 15 g/L and 2 g/250 mL Erlenmeyer flask; respectively).

### Model accuracy checking

**Fig. 6:**
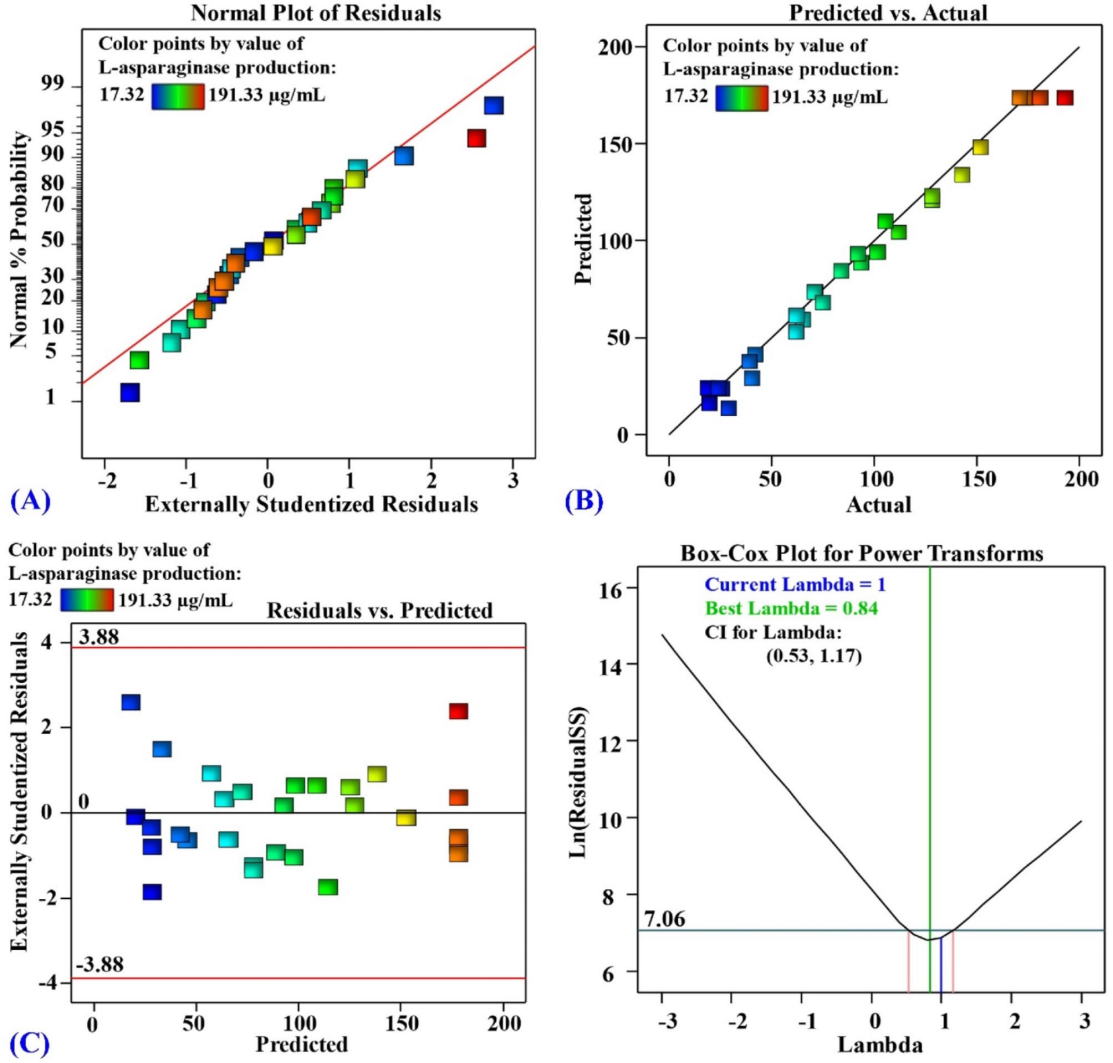
(A) NPP of the residuals, (B) a graphic showing the actual versus predicted, (C) plot of residuals versus predicted and (D) Box- Cox plot for power transforms.

The statistical analysis was performed to validate the precision of the design. Figure 6A shows the normal probability plot (NPP) of the residuals. NPP is a crucial diagnostic tool used to assess if the residuals follow to a normal distribution. The residuals data points should be arranged in close proximity to a diagonal line and relatively uniformly dispersed. The data points for the residuals exhibited a normal distribution, located along the diagonal line for predicted L-asparaginase production using *S. violaceoruber*, confirming the validation of the model. Figure 6B displays a graph comparing the predicted and actual L-asparaginase production. The dots are gathered around the line of best fit on the graph, demonstrating that the experimental and the predicted L-asparaginase production are highly correlated^3^. Figure 6C displays a graph illustrating the residuals versus the predicted L-asparaginase production. Figure 6C indicates that the residuals have a consistent variance. The model is valid because the residuals follow a uniform and random distribution, uniformly distributed both up and down the zero line, with no apparent pattern^55^.

The Box-Cox graph is illustrated in Fig. 6D. The blue line illustrates the current transformation (Lambda = 1). The green line illustrates the optimal lambda value (0.84). The red lines display the lowest and highest values of the confidence intervals, which range from 0.53 to 1.17. The model is in the optimal zone because the blue line, which represents the current transformation, is located between the two red lines, which represent the minimum and maximum values of the confidence intervals. Therefore, there is no demand for transformation of the data as the model accurately correlates to the experimental results^56^.

### ANN modeling prediction for L-asparaginase production

Artificial Neural Networks (ANN) are an innovative approach in artificial intelligence that enables the creation of reliable computational models to interpret and analyze data in a way that resembles human brain processes^16^. The production of L-asparaginase by *Aspergillus terreus* MTCC 1782 was predicted using an artificial neural network (ANN) strategy^57^. The ANN model achieved better results compared to the response surface methodology (RSM). Two factors significantly influence the topology or construction of ANN: the numbers of hidden layer neurons or nodes and the overall number of layers. A standard neural network design consists of three layers: input, hidden, and output. These layers are made up of interconnected artificial neurons^58^.

**Fig. 7:**
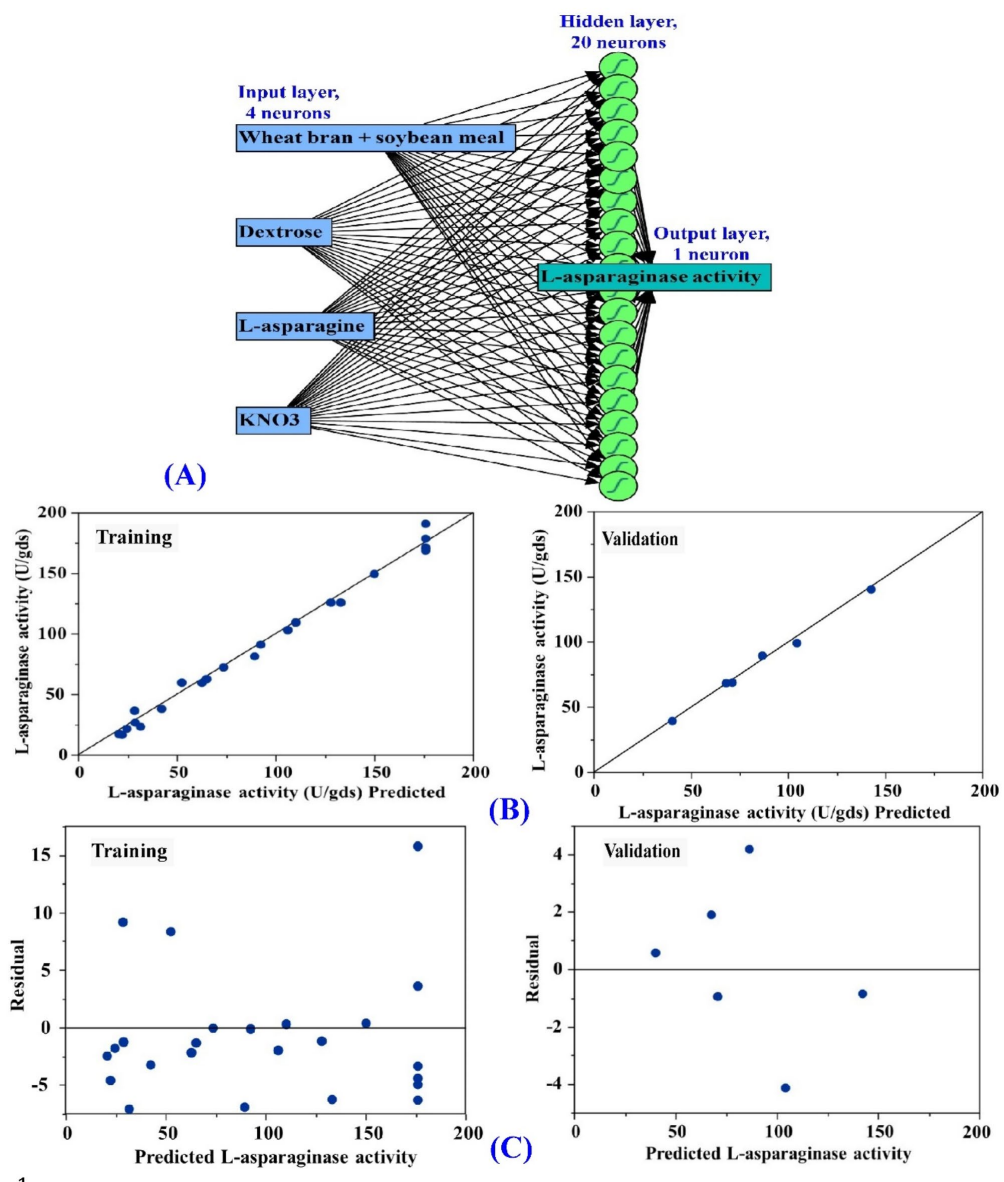
ANN for L-asparaginase production by *S. violaceoruber* (A), predicted versus actual (B), predicted versus the residuals of training and validation processes for production of L-asparaginase (C).

The network architecture of ANN modeling comprises the validation of the final ANN model in addition to the training and learning processes^59^. In order to optimize the ANN’s performance in this investigation, the following parameter adjustments were made: The model NTanH (20), the number of tours (5000), the learning rate (0.1), and the validation technique (holdback, 0.2) were all applied. Initial data for four independent variables including soybean and wheat bran mixture in a ratio of 1:1; w/w (X_1_), dextrose (X_2_), L-asparagine (X_3_), and KNO_3_ (X_4_) are collected by the input layer. The hidden layer, which serves as a link between the input and output layers, has twenty neurons (Fig. 7A). A series of mathematical operations is performed on the data that is provided via the input layer by hidden layer neurons to generate output at the output layer. The number of neurons in the hidden layer influences the prediction accuracy of ANN^60^. The ANN analysis and model performance in terms of its ability to predict extracellular L-asparaginase production by *S. violaceoruber* are illustrated in Table 6.

### Evaluation of an ANN model

ANN was employed to predict the production of L-asparaginase using the *S. violaceoruber*. The predictions were based on the experimental data, which are presented in Table 3. Comparison of the experimental results for L-asparaginase production against the predictions of the ANN model is illustrated in Fig. 7B. The data points are closely organized around the best prediction line in both the training and validation procedures, suggesting the model’s reliability. Furthermore, the residual data points exhibit a symmetrical normal distribution, wherein an equivalent number of points are situated on each side of the regression line (Fig. 7C). This demonstrates the appropriateness of the ANN model.

### The comparative predictive capability of ANN and CCD

In order to achieve maximum L-asparaginase production, it was necessary to find the optimum value for each factor through the use of prediction models like CCD or ANN. Compared to the CCD, the ANN-predicted values of L-asparaginase production had smaller residuals and showed better agreement with the experimental data (Table 3). JMP Pro14’s model comparison tab was applied to assess the precision of the CCD and ANN predictions and identify the most appropriate model based on their predictions for L-asparaginase production and the corresponding experimental data. The following metrics were used to compare the predictive power of the ANN and CCD models: mean absolute deviation (MAD), R^2^ value, sum of squared errors (SSE), and root mean square error (RMSE)^17,61^. In a comparison of the predictive capabilities of ANN and CCD, it is evident that ANN demonstrates a substantially higher level of accuracy (Table 6). Table 6 shows that ANN has a higher R^2^ value of 0.9916, whereas RASE and AAE have lesser values of 4.96 and 3.62, respectively. Consequently, ANN is more capable of predicting the optimal value of each factor to maximize the production of L-asparaginase. The improved predictive capability of the ANN that has been observed can be attributable to the function of repeatedly training the neurons for a range of different independent factors^16^.

**Table 6:** ANN analysis and comparison of overall model performance of the ANN and CCD models in terms of their ability to predict extracellular L-asparaginase production by *S. violaceoruber*.

Measure	ANN		Overall model performance		
	Training	Validation	Statistics	Measures of Fit for CCD	Measures of Fit for ANN
R^**2**^	0.9915	0.9932	R^2^	0.9891	0.9916
RMSE	5.40	2.59	RASE	5.67	4.96
MAD	4.00	2.10	AAE	4.64	3.62
SSE	698.94	40.15	Freq	30	30
Sum Freq	24	6			

SSE: the sum of squares error; AAE: the average absolute error MAD: mean absolute deviation; RASE: root average squared error; RMSE: root mean squared error.

### L-asparaginase production predictions using the desirability function (DF)

**Fig. 8:**
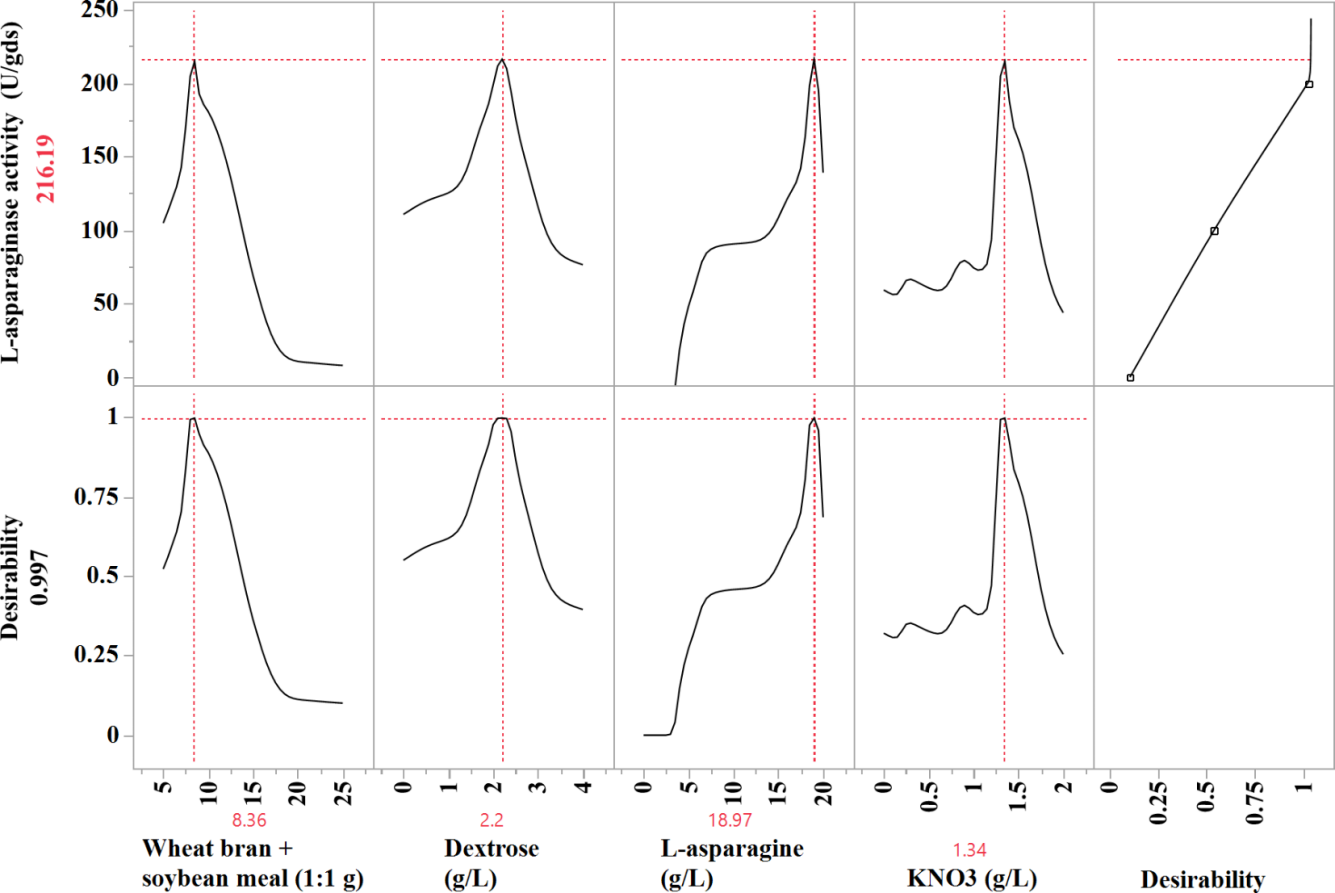
The optimization plot illustrates the optimal predicted values of the process factors for maximal predicted L-asparaginase production by *S. violaceoruber*, along with the desirability function.

In order to maximize L-asparaginase production, the optimal predicted conditions have been determined using the desirability function (as illustrated in Fig. 8). The JMP Pro14 software’s desirability function can be set to any value between 0 (representing undesirability) and 1(representing desirability). Prior to experimental verification of the optimization process, the desirability function’s value is usually estimated mathematically. The maximal predicted L-asparaginase production using *S. violaceoruber* was determined using the desirability function to be 216.19 U/gds. The optimal predicted conditions resulted in the highest predicted L-asparaginase production were 8.46 g/250 mL Erlenmeyer flask of soybean and wheat bran mixture (1:1; w/w), 2.2 g/L of dextrose, 18.97 g/L of L-asparagine, and 1.34 g/L of KNO_3_. The desirability value for these conditions was 0. 997. The verification revealed that the ANN was highly accurate and predictive, as the experimental values (207.55 U/gds) approximately matched the theoretical values.

## Conclusion

The current study clearly proved that soils are a rich source of actinomycetes that produce L-asparaginase. *S. violaceoruber*, isolated from soil, has a high capacity for producing L-asparaginase. The production of L-asparaginase using *S. violaceoruber* has been analyzed, validated, and predicted using both CCD and ANN. Upon comparing the predictive capacities of ANN with CCD, it can be concluded that ANN is more effective than CCD in predicting the optimal value of each factor for maximizing the production of L-asparaginase.

## Data Availability

All data generated or analyzed during this study are included in this article.

## References

[1] Castro, D. et al. L-asparaginase production review: bioprocess design and biochemical characteristics. *Appl. Microbiol. Biotechnol.* **105**, 4515–4534 (2021). 34059941 10.1007/s00253-021-11359-y

[2] El-Naggar, N. E. Extracellular production of the oncolytic enzyme, L-asparaginase, by newly isolated *Streptomyces* sp. strain NEAE-95 as potential microbial cell factories: Optimization of culture conditions using response surface methodology. *Curr. Pharm. Biotechnol.* **16**, 162–178 (2015). 25395212 10.2174/1389201015666141113123910

[3] El-Naggar, N. E. et al. Process development for scale-up production of a therapeutic L-asparaginase by S*treptomyces brollosae* NEAE-115 from shake flasks to bioreactor. *Sci. Rep.* **9**, 13571 (2019). 31537817 10.1038/s41598-019-49709-6 PMC6753079

[4] Chergui, A. et al. Optimization of intracellular L-Asparaginase production by *Streptomyces paulus* CA01 isolated from wheat bran using the response surface methodology. *Biotechnol. Biotechnol. Equip.* **37**, 2281493 (2023).

[5] El-Naggar, N. E. & El-Shweihy, N. M. Bioprocess development for L-asparaginase production by *Streptomyces rochei*, purification and in-vitro efficacy against various human carcinoma cell lines. *Sci. Rep.* **10**, 7942 (2020). 32409719 10.1038/s41598-020-64052-x PMC7224186

[6] El-Gendy, M. M. A. A., Awad, M. F., El-Shenawy, F. S. & El-Bondkly, A. M. A. Production, purification, characterization, antioxidant and antiproliferative activities of extracellular L-asparaginase produced by *Fusarium equiseti* AHMF4. *Saudi J. Biol. Sci.* **28**, 2540–2548 (2021). 33911966 10.1016/j.sjbs.2021.01.058 PMC8071902

[7] El-Naggar, N. E., El-Ewasy, S. M. & El-Shweihy, N. M. Microbial L-asparaginase as a potential therapeutic agent for the treatment of acute lymphoblastic leukemia: the pros and cons. *Int. J. Pharmacol.* **10**, 182–199 (2014).

[8] Duval, M. et al. Comparison of *Escherichia coli*–asparaginase with *Erwinia-asparaginase* in the treatment of childhood lymphoid malignancies: results of a randomized European Organisation for Research and Treatment of Cancer—Children’s Leukemia Group phase 3 trial. *Blood J. Am. Soc. Hematol.* **99**, 2734–2739 (2002). 10.1182/blood.v99.8.2734 11929760

[9] Dias, F. F. G., Ruiz, A. L. T. G., Della Torre, A. & Sato, H. H. Purification, characterization and antiproliferative activity of L-asparaginase from *Aspergillus oryzae* CCT 3940 with no glutaminase activity. *Asian Pac. J. Trop. Biomed.* **6**, 785–794 (2016).

[10] Thomas, X., Cannas, G., Chelghoum, Y. & Gougounon, A. Therapeutic alternatives to native L-asparaginase in the treatment of adult acute lymphoblastic leukemia. *Bull. Cancer* **97**, 1105–1117 (2010). 20693115 10.1684/bdc.2010.1168

[11] El-Ghonemy, D. H., Ali, S. A., Abdel-Megeed, R. M. & Elshafei, A. M. Therapeutic impact of purified Trichoderma viride L-asparaginase in murine model of liver cancer and in vitro Hep-G2 cell line. *J. Genet. Eng. Biotechnol.* **21**, 38 (2023). 36995465 10.1186/s43141-023-00493-x PMC10063745

[12] El-Naggar, N. E. A. *Streptomyces*-basedcell factories for production of biomolecules and bioactive metabolites. In *MicrobialCell Factories Engineering for Production of Biomolecules* (pp. 183-234). Academic Press. (2021).

[13] El-Bessoumy, A. A., Sarhan, M. & Mansour, J. Production, isolation, and purification of L-asparaginase from *Pseudomonas aeruginosa* 50071 using solid-state fermentation. *BMB Rep.* **37**, 387–393 (2004). 10.5483/bmbrep.2004.37.4.387 15469724

[14] Kumar, V. et al. Recent developments on solid-state fermentation for production of microbial secondary metabolites: Challenges and solutions. *Bioresour. Technol.* **323**, 124566 (2021). 33390315 10.1016/j.biortech.2020.124566

[15] Sherief, A. A., El-Naggar, N. E. & Hamza, S. S. Bioprocessing of lignocellulosic biomass for production of bioethanol using thermotolerant *Aspergillus fumigatus* under solid state fermentation conditions. *Biotechnology* **9**, 513–522 (2010).

[16] El-Naggar, N. E., Dalal, S. R., Zweil, A. M. & Eltarahony, M. Artificial intelligence-based optimization for chitosan nanoparticles biosynthesis, characterization and in–vitro assessment of its anti-biofilm potentiality. *Sci. Rep.* **13**, 4401 (2023). 36928367 10.1038/s41598-023-30911-6 PMC10019797

[17] El-Naggar, N. E., Bashir, S. I., Rabei, N. H. & Saber, W. I. A. Innovative biosynthesis, artificial intelligence-based optimization, and characterization of chitosan nanoparticles by *Streptomyces microflavus* and their inhibitory potential against *Pectobacterium carotovorum*. *Sci. Rep.* **12**, 21851 (2022). 36528632 10.1038/s41598-022-25726-w PMC9759534

[18] Gulati, R., Saxena, R. K. & Gupta, R. A rapid plate assay for screening l-asparaginase producing micro‐organisms. *Lett. Appl. Microbiol.* **24**, 23–26 (1997). 9024001 10.1046/j.1472-765x.1997.00331.x

[19] Lingappa, K. & Babu, C. S. V. Production of lovastatin by solid state fermentation of carob (*Ceratonia siliqua*) pods using *Aspergillus terreus*. *KLVB* **2005**, 28 (2005).

[20] Wriston, J. C. Jr & Yellin, T. O. L-asparaginase: a review. *Adv. Enzymol. Relat. Areas Mol. Biol.* **39**, 185–248 (1973). 4583638 10.1002/9780470122846.ch3

[21] Imada, A., Igarasi, S., Nakahama, K. & Isono, M. Asparaginase and glutaminase activities of micro-organisms. *Microbiology* **76**, 85–99 (1973). 10.1099/00221287-76-1-85 4723072

[22] Shirling, E. B. T. & Gottlieb, D. Methods for characterization of *Streptomyces* species. *Int. J. Syst. Bacteriol.* **16**, 313–340 (1966).

[23] El-Naggar, N. E., Sherief, A. A. & Hamza, S. S. *Streptomyces aegyptia* NEAE 102, a novel cellulolytic streptomycete isolated from soil in Egypt. *Afr. J. Microbiol. Res.* **5**, 5308–5315 (2011).

[24] El-Naggar, N. E. & Abdelwahed, N. A. M. Optimization of process parameters for the production of alkali-tolerant carboxymethyl cellulase by newly isolated *Streptomyces* sp. strain NEAE-D. *Afr. J. Biotechnol.* **11**, 1185–1196 (2012).

[25] El-Naggar, N. E. & Hamouda, R. Antimicrobial potentialities of *Streptomyces lienomycini* NEAE-31 against human pathogen multidrug-resistant *Pseudomonas aeruginosa*. *Int. J. Pharmacol.* **12**, 769–788 (2016).

[26] Li, Q., Chen, X., Jiang, Y. & Jiang, C. Cultural, physiological, and biochemical identification of actinobacteria. *Actinobacteria-Basics Biotechnol. Appl.* **2016**, 87–111 (2016).

[27] Sambrook, J., Fritsch, E. F. & Maniatis, T. *Molecular Cloning: A Laboratory Manual* 31–39 (Cold Spring Harbor laboratory press, 1989).

[28] El-Naggar, N. E. Isolation,screening and identification of actinobacteria with uricase activity: statistical optimization of fermentation conditions for improved production ofuricase by *Streptomyces rochei* NEAE–25. *Int. J. Pharmacol. ***11**(7), 644-658 (2015).26728027

[29] Altschul, S. F. et al. Gapped BLAST and PSI-BLAST: a new generation of protein database search programs. *Nucleic Acids Res.* **25**, 3389–3402 (1997). 9254694 10.1093/nar/25.17.3389 PMC146917

[30] Tamura, K., Stecher, G. & Kumar, S. MEGA11: molecular evolutionary genetics analysis version 11. *Mol. Biol. Evol.* **38**, 3022–3027 (2021). 33892491 10.1093/molbev/msab120 PMC8233496

[31] El-Naggar, N. E. & Moawad, H. *Streptomyces brollosae* sp. nov., NEAE-115, a novel L-asparaginase producing actinomycete isolated from Brollos Lake at the Mediterranean coast of Egypt. *J. Pure Appl. Microbiol.* **9**, 11–20 (2015).

[32] Soliman, H. M., El-Naggar, N. E. & El-Ewasy, S. M. Bioprocess optimization for enhanced production of L-asparaginase via two model-based experimental designs by alkaliphilic *Streptomyces fradiae* NEAE-82. *Curr. Biotechnol.* **9**, 23–37 (2020).

[33] Parte, A. et al. *Bergey’s Manual of Systematic Bacteriology: Volume 5: the Actinobacteria* (Springer Science & Business Media, 2012).

[34] Saitou, N. & Nei, M. The neighbor-joining method: a new method for reconstructing phylogenetic trees. *Mol. Biol. Evol.* **4**, 406–425 (1987). 3447015 10.1093/oxfordjournals.molbev.a040454

[35] El-Naggar, N. E., Moawad, H. & Abdelwahed, N. A. M. Optimization of fermentation conditions for enhancing extracellular production of L-asparaginase, an anti-leukemic agent, by newly isolated *Streptomyces brollosae* NEAE-115 using solid state fermentation. *Ann. Microbiol.* **67**, 1–15 (2017).

[36] Isaac, G. S. & Abu-Tahon, M. A. Production of extracellular anti-leukemic enzyme L-asparaginase from *Fusarium solani* AUMC 8615 grown under solid-state fermentation conditions: purification and characterization of the free and immobilized enzyme. *Egypt. J. Bot.* **56**, 799–816 (2016).

[37] El-Naggar, N. E., Saber, W. I. A., Zweil, A. M. & Bashir, S. I. An innovative green synthesis approach of chitosan nanoparticles and their inhibitory activity against phytopathogenic Botrytis cinerea on strawberry leaves. *Sci. Rep.* **12**, 3515 (2022). 35241695 10.1038/s41598-022-07073-y PMC8894456

[38] El-Naggar, N. E., Hamouda, R. A., El-Khateeb, A. Y. & Rabei, N. H. Biosorption of cationic Hg^2+^ and Remazol brilliant blue anionic dye from binary solution using *Gelidium corneum* biomass. *Sci. Rep.* **11**, 20908 (2021). 34686690 10.1038/s41598-021-00158-0 PMC8536736

[39] Edwinoliver, N. G. et al. Scale up of a novel tri-substrate fermentation for enhanced production of *Aspergillus niger* lipase for tallow hydrolysis. *Bioresour. Technol.* **101**, 6791–6796 (2010). 20400303 10.1016/j.biortech.2010.03.091

[40] de Oliveira, R. L., da Silva, M. F., Converti, A. & Porto, T. S. Production of β-fructofuranosidase with transfructosylating activity by *Aspergillus tamarii* URM4634 Solid-State Fermentation on agroindustrial by-products. *Int. J. Biol. Macromol.* **144**, 343–350 (2020). 31838073 10.1016/j.ijbiomac.2019.12.084

[41] de Menezes, L. H. S. et al. Application of a constrained mixture design for lipase production by *Penicillium roqueforti* ATCC 10110 under solid-state fermentation and using agro-industrial wastes as substrate. *Prep. Biochem. Biotechnol.* **52**, 885–893 (2022). 34965202 10.1080/10826068.2021.2004547

[42] Sharma, D. & Mishra, A. Synergistic effects of ternary mixture formulation and process parameters optimization in a sequential approach for enhanced L-asparaginase production using agro-industrial wastes. *Environ. Sci. Pollut Res.* **31**, 17858–17873 (2024). 10.1007/s11356-023-26977-4 37086318

[43] Dharmsthiti, S. C. & Luechai, S. Purification and characterization of asparaginase from solid state culture of *Aspergillus niger* AK10. *Int. J. Biotechnol. Biochem.* **7**, 81–91 (2011).

[44] El-Naggar, N. E., Moawad, H., El-Shweihy, N. M. & El-Ewasy, S. M. Optimization of culture conditions for production of the anti-leukemic glutaminase free L-asparaginase by newly isolated *Streptomyces olivaceus* NEAE-119 using response surface methodology. *Biomed Res. Int.* **2015**, 458 (2015). 10.1155/2015/627031 PMC4477217 26180806

[45] Vimal, A. & Kumar, A. Biotechnological production and practical application of L-asparaginase enzyme. *Biotechnol. Genet. Eng. Rev.* **33**, 40–61 (2017). 28766374 10.1080/02648725.2017.1357294

[46] Vala, A. K. et al. Characterization of L-asparaginase from marine-derived *Aspergillus niger* AKV-MKBU, its antiproliferative activity and bench scale production using industrial waste. *Int. J. Biol. Macromol.* **108**, 41–46 (2018). 29175524 10.1016/j.ijbiomac.2017.11.114

[47] Abdel-Hamid, N. S., Abdel-Khalek, H. H., Ramadan, E., Mattar, Z. A. & Abou-Taleb, K. A. Optimization of L-asparaginase production from *Fusarium oxysporum* F-S3 using irradiated pomegranate peel under solid-state fermentation. *Egypt. J. Chem.* **65**, 381–397 (2022).

[48] Baskar, G. & Renganathan, S. Optimization of media components and operating conditions for exogenous production of fungal L-asparaginase. *Chiang Mai J. Sci.* **38**, 270–279 (2011).

[49] El-Naggar, N. E., Rabei, N. H. & El-Malkey, S. E. Eco-friendly approach for biosorption of Pb^2+^ and carcinogenic Congo red dye from binary solution onto sustainable *Ulva lactuca* biomass. *Sci. Rep.* **10**, 16021 (2020). 32994453 10.1038/s41598-020-73031-1 PMC7525567

[50] Alrumman, S. A. et al. Production and anticancer activity of an L-asparaginase from *Bacillus licheniformis* isolated from the Red Sea, Saudi Arabia. *Sci. Rep.* **9**, 3756 (2019). 30842557 10.1038/s41598-019-40512-x PMC6403232

[51] Kumar, S., Dasu, V. V. & Pakshirajan, K. Localization and production of novel L-asparaginase from *Pectobacterium carotovorum* MTCC 1428. *Process. Biochem.* **45**, 223–229 (2010).

[52] Warangkar, S. C. & Khobragade, C. N. Screening, enrichment and media optimization for L-asparaginase production. *J. cell. tissue Res.* **9**, 1963 (2009).

[53] Geckil, H. & Gencer, S. Production of L-asparaginase in *Enterobacter aerogenes* expressing Vitreoscilla hemoglobin for efficient oxygen uptake. *Appl. Microbiol. Biotechnol.* **63**, 691–697 (2004). 14593509 10.1007/s00253-003-1482-5

[54] Garaev, M. M. & Golub, E. I. Mechanism of action of glucose on L-asparaginase synthesis by *Escherichia coli* bacteria. *Mikrobiologiia* **46**, 433–439 (1977). 197380

[55] Hamouda, R. A., El-Naggar, N. E. A., Doleib, N. M., & Saddiq, A. A. Bioprocessing strategies for cost-effective simultaneous removal of chromium and malachite green by marine alga *Enteromorpha intestinalis*. *Scientific Reports. ***10**(1), 13479 (2020). 32778759 10.1038/s41598-020-70251-3 PMC7417574

[56] El-Sawah, A. A., El-Naggar, N. E., Eldegla, H. E. & Soliman, H. M. Bionanofactory for green synthesis of collagen nanoparticles, characterization, optimization, in-vitro and in-vivo anticancer activities. *Sci. Rep.* **14**, 6328 (2024). 38491042 10.1038/s41598-024-56064-8 PMC10943001

[57] Gurunathan, B. & Sahadevan, R. Optimization of culture conditions and bench-scale production of L-asparaginase by submerged fermentation of *Aspergillus terreus* MTCC 1782. *J. Microbiol. Biotechnol.* **22**, 923–929 (2012). 22580311 10.4014/jmb.1112.12002

[58] El-Naggar, N. E. et al. Process optimization for gold nanoparticles biosynthesis by *Streptomyces albogriseolus* using artificial neural network, characterization and antitumor activities. *Sci. Rep.* **14**, 4581 (2024). 38403677 10.1038/s41598-024-54698-2 PMC10894868

[59] El-Naggar, N. E. et al. Artificial neural network approach for prediction of AuNPs biosynthesis by *Streptomyces flavolimosus*, characterization, antitumor potency in-vitro and in-vivo against Ehrlich ascites carcinoma. *Sci. Rep.* **13**, 12686 (2023). 37542154 10.1038/s41598-023-39177-4 PMC10403537

[60] Nidhul, K., Thummar, D., Yadav, A. K. & Anish, S. Machine learning approach for optimization and performance prediction of triangular duct solar air heater: A comprehensive review. *Sol Energy. ***255**, 396–415 (2023).

[61] Saber, W. I. A. et al. Rotatable central composite design versus artificial neural network for modeling biosorption of Cr^6+^ by the immobilized *Pseudomonas alcaliphila* NEWG-2. *Sci. Rep.* **11**, 1717 (2021). 33462359 10.1038/s41598-021-81348-8 PMC7814044

